# Tooth Autotransplantation, Autogenous Dentin Graft, and Growth Factors Application: A Method for Preserving the Alveolar Ridge in Cases of Severe Infraocclusion—A Case Report and Literature Review

**DOI:** 10.3390/jcm13133902

**Published:** 2024-07-03

**Authors:** Paulina Adamska, Dorota Pylińska-Dąbrowska, Marcin Stasiak, Hanna Sobczak-Zagalska, Antoni Jusyk, Adam Zedler, Michał Studniarek

**Affiliations:** 1 Division of Oral Surgery, Faculty of Medicine, Medical University of Gdańsk, 7 Dębinki Street, 80-210 Gdańsk, Poland; adam.zedler@gumed.edu.pl; 2 Department of Dental Prosthetics, Faculty of Medicine, Medical University of Gdańsk, 18 Orzeszkowej Street, 80-204 Gdańsk, Poland; dorota.pylinska-dabrowska@gumed.edu.pl; 3 Division of Orthodontics, Faculty of Medicine, Medical University of Gdańsk, 42c Aleja Zwycięstwa, 80-210 Gdańsk, Poland; marcin.stasiak@gumed.edu.pl; 4 Department of Pediatric Dentistry, Faculty of Medicine, Medical University of Gdańsk, 18 Orzeszkowej Street, 80-204 Gdańsk, Poland; hanna.sobczak-zagalska@gumed.edu.pl; 5 University Dental Center, Medical University of Gdańsk, 1a Dębowa Street, 80-204 Gdańsk, Poland; antoni.jusyk@gumed.edu.pl; 6 Department of Radiology, Faculty of Medicine, Medical University of Gdańsk, 17 Smoluchowskiego Street, 80-210 Gdańsk, Poland; michal.studniarek@gumed.edu.pl

**Keywords:** autografting, autologous, autotransplantation, bone regeneration, growth factor, plasma, platelet-rich fibrin

## Abstract

**Background**: Tooth infraocclusion is a process in which a completely or partially erupted tooth gradually moves away from the occlusal plane. Submerged teeth can lead to serious complications. Treating teeth with infraocclusion is very challenging. One of the procedures allowing for the replacement of a missing tooth is autotransplantation. The aim of this paper is to review the literature on teeth autotransplantation, supported by a case report involving the autotransplantation of a third mandibular molar into the site of an extracted infraoccluded first mandibular molar, as well as the utilization of advanced platelet-rich fibrin (A-PRF) alongside autogenous dentin grafts for bone tissue regeneration. **Methods**: A severely infraoccluded first permanent right mandibular molar was extracted and then ground to obtain the dentin graft. A-PRF clots (collected from the patient’s peripheral blood) were added to the autogenous dentin graft, to create the A-PRF membrane. An atraumatic extraction of the lower left third molar was performed and then it was transplanted into the socket of tooth no. 46. Immediately after transplantation, tooth no. 38 was stabilized with orthodontic bracket splints for 3 months. The patient attended regular follow-up visits within 12 months. **Results**: After one year, the patient did not report any pain. In the clinical examination, the tooth and surrounding tissues did not show any signs of infection. However, radiographically, cervical inflammatory resorption, unchanged pulp canal dimensions, absent root growth, periapical radiolucency, and lack of apical and marginal healing were observed. Reconstruction of the bone defect was obtained and the alveolar ridge of the mandible was preserved. Due to poor stability of the tooth and severe resorption, the tooth needed to be extracted. **Conclusions**: This study is designed to critically evaluate the efficacy of autotransplantation, the application of growth factors, and the integration of autogenous dentin grafts in remedying dental deficiencies resulting from reinclusion. We aim to point out the possible causes of treatment failure.

## 1. Introduction

Tooth infraocclusion is a dynamic process where a tooth, whether it has completely or partially erupted, progressively diverges from the alignment of fully erupted teeth, distancing itself from the occlusal plane. A tooth is submerged when its intact marginal ridges are situated more than 0.5 mm beneath the intact marginal ridges of the adjacent, normally erupted teeth. Without early diagnosis and proper treatment, an infraoccluded tooth can be completely covered with the gingiva and deeply immersed in the alveolar bone. Immersed teeth can occur in both deciduous and permanent dentition [1,2,3]. This disorder predominantly affects the first deciduous molar in the mandible. The occurrence of a submerged milk tooth may be linked to the absence of the germ for the corresponding permanent tooth. In the case of permanent teeth, the most frequent instances of reinclusion primarily involve the first molars in the mandible, with the second molars being less commonly affected. Dilaceration of the root may occur in the mandible due to its formation in contact with the cortical bone. A familiar relationship has been observed in the occurrence of this disorder. Patients who have developed this condition within one tooth are often affected by this pathology in other teeth. The incidence of this phenomenon varies from 1.3% to 38.5% [1,2,3,4,5,6]. 

Submerged teeth can cause gingiva inflammation and result in periodontal disease formation. They can form abscesses, caused by tooth decay (the tooth is connected to the oral environment by the epithelial duct). A reincluded deciduous tooth may also inhibit the growth of the permanent tooth germ. In advanced cases, teeth can sink to the bottom of the sinus and inferior alveolar nerve canal (IANC). Additionally, reincluded teeth adversely affect the occlusion and development of the entire stomatognathic system, manifesting through the underdevelopment of the alveolar process, supraocclusion of opposing teeth, and the inclination of adjacent teeth. This condition can lead to a decrease in space and a reduction in the dental arch. Submerged teeth are often qualified for extraction due to associated inflammation. The extraction of an infraoccluded tooth, which often develops ankylosis, is a difficult surgical procedure. Further, some complicated extractions may lead to postoperative complications that may influence recovery, such as alveolar osteitis [7,8,9]. This can lead to extensive oral tissue loss, and in such cases, the patient needs an urgent procedure to replace lost tissues. Infraocclusion is classified as the fifth stage by IOTN (the Index of Orthodontic Treatment Need) as a disorder requiring orthodontic treatment [3,4]. Premature removal of a reincluded tooth due to the significant severity of the pathology is a therapeutic challenge. Implant treatment should not be performed in patients of developmental age due to ongoing bone growth and subsequent consequences.

There are few treatment options for restoring a missing tooth. They include prosthetic appliances like bridges, implants, removable dentures, and orthodontic space closures. Another alternative, which is often overlooked, is autotransplantation, which is a surgical procedure that involves repositioning a tooth from one site to another within the same individual. It is also known as controlled, sterile avulsion and reimplantation of a tooth into a different site in the same person [6]. The eruption of the autotransplanted tooth could stimulate vertical bone growth and, thus, the reconstruction of the bone defect [10].

Platelet-rich preparations are autogenous products obtained from the patient’s blood. One preparation is advanced platelet-rich fibrin (A-PRF). A-PRF is widely acknowledged for its pivotal role in enhancing healing processes in both hard and soft tissues, including bone augmentation, angiogenesis, wound healing, and periodontal regeneration [11,12,13,14,15,16,17,18,19,20,21,22,23,24,25,26]. 

The grafts prepared from extracted teeth have been described as alternative bone grafts. Autogenous tooth bone graft material (ATBG, also called autogenous dentine graft, ADG), such as density, homogeneity, roughness, and its physiochemical features (e.g., calcium/phosphate ion dissolution) are similar to human cortical bone characteristics [27]. ATBG has been shown to be a safe and biocompatible material with high bone formation activity that accelerates bone healing because of its osteoinductive properties [28,29,30,31,32,33]. Filling the defect area with a “sticky tooth” and covering it with an A-PRF membrane accelerates bone formation and wound healing. A sticky tooth has its own body and can be easily molded into the required shape, thus offering easy handling and preventing dispersion. The use of the A-PRF membrane as a barrier membrane over the sticky tooth and I-PRF increases new bone formation. This is due to the availability of an environment enriched with growth factors, which promotes and has a positive effect on tissue healing [23,26].

To the authors’ knowledge, this is the first article about tooth autotransplantation and the use of advanced platelet-rich fibrin and autogenous dentin graft in bone tissue regeneration. The aim of the study is to evaluate the roles of autotransplantation, growth factors, and autogenous dentin graft in treating the lack of a tooth due to reinclusion.

## 2. Case Presentation

A 21-year-old patient was referred to the Division of Oral Surgery at the Medical University of Gdańsk for the management of severe infraocclusion of the lower right first molar (tooth no. 46, according to the FDI fr. *Federation Dentaire Internationale*). Despite undergoing orthodontic treatment for two years, attempts at orthodontic extrusion to correct the tooth’s position were unsuccessful. The tooth continued to undergo reinclusion, indicating a progressive worsening of its infraocclusion, despite therapeutic efforts.

**Figure 1:**
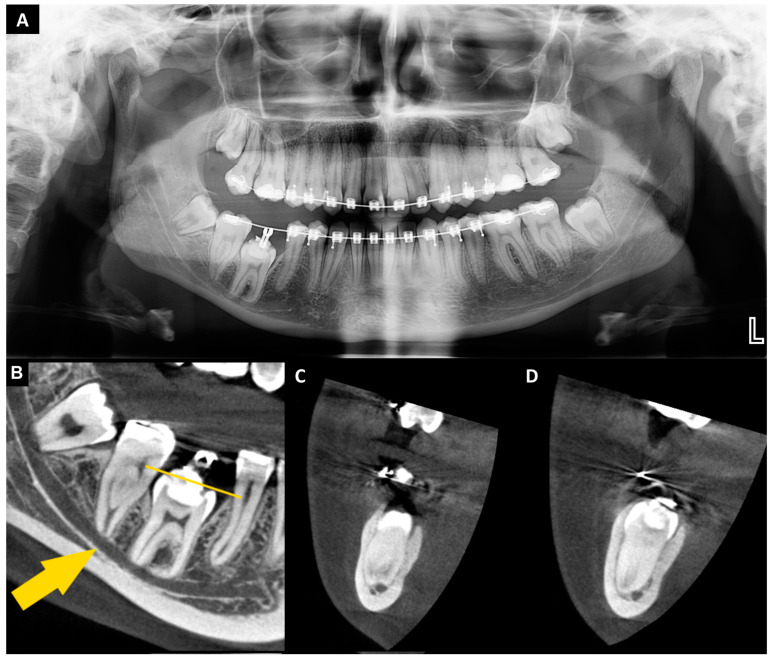
Orthopantomography (**A**) reincluded tooth no. 46 and donor—tooth no. 38; cone beam computed tomography (**B**–**D**) (radiological examinations performed 1 month before the procedure): (**B**) sagittal view—severe reinclusion of tooth no. 46 (yellow line—the crown of the tooth located under the mucous membrane); yellow arrow—inferior alveolar nerve canal); (**C**) cross-sectional view—mesial root of tooth no. 46 in relation to IANC; (**D**) cross-sectional view—distal root of tooth no. 46 in relation to IANC.

The patient was generally healthy, with no systemic diseases or allergies, did not take any medications, was a non-smoker, and had good oral hygiene. Extraoral and intraoral examinations, orthopantomography, and cone beam computed tomography (CBCT) were performed (Figure 1A–D). The CBCT was assessed using CS 3D Imaging v3.5.18 software (Carestream Health Inc., Trophy, Croissy-Beaubourg, France). The imaging conditions were 84 kV, 5 mA, with a voxel size of 0.1 mm, a field of view (FOV) of 6×6 cm, and CTDIvol 2.66 mGy. The CBCT examination revealed that tooth no. 46 was located under the mucous membrane while maintaining contact with the oral environment through a narrow canal lined with epithelium. This tooth was classified as a severe type of reinclusion. The mesial root was dilacerated and both of the roots were in close position to the inferior alveolar nerve canal. Removing this tooth was associated with a high risk of nerve damage and neurosensory disturbances. Due to the presence of severe infraocclusion of tooth no. 46, the surgical procedure was planned as follows (after 2 years of unsuccessful orthodontic treatment, without prior surgical support): the extraction of tooth no. 46, grinding the tooth no. 46 to obtain a dentin graft, adding A-PRF clots (collected from the patient’s peripheral blood) to the autogenous dentin graft, creating the A-PRF membrane, and atraumatic extraction and transplantation of tooth no. 38 (lower left third molar). The alveolar socket after the removal of tooth no. 46 did not require additional preparation due to the larger size of tooth no. 46 compared to tooth no. 38. Stabilization of tooth no. 38 was performed with an orthodontic bracket splint, and a bone graft was put in place of tooth no. 46. The patient signed an informed written consent for the procedures and the use of the data and photos for publication. 

**Figure 2:**
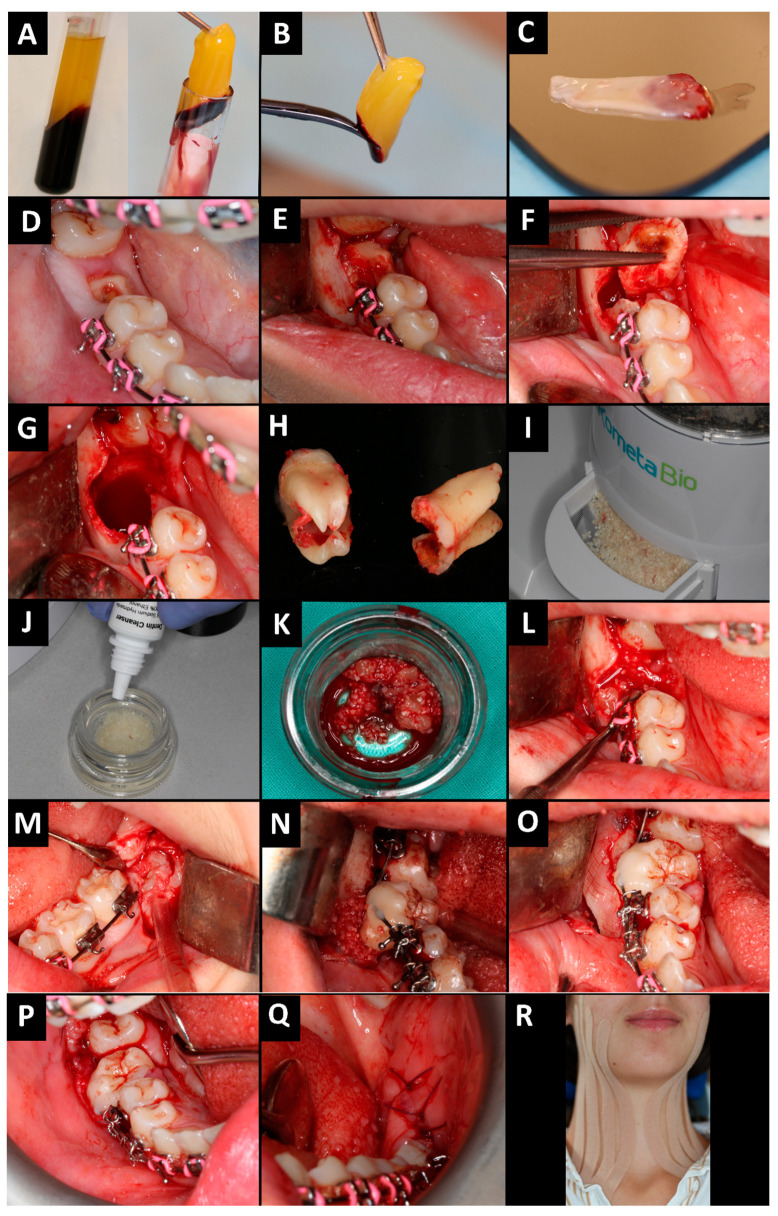
(**A**) A-PRF in glass-coated plastic tubes; (**B**) A-PRF clot was dissected by scissors from the red blood cell base at the bottom; (**C**) A-PRF membrane preparation; (**D**) initial situation; (**E**) envelope flap preparation; (**F**) osteotomy, tooth separation, and extraction; (**G**) empty alveolar socket; (**H**) removed tooth no. 46; (**I**) tooth grinding; (**J**) ADG procession; (**K**) mixing ADG with A-PRF; (**L**) filling the socket with ADG and A-PRF; (**M**) minimally invasive extraction of tooth no. 38; (**N**) tooth no. 38 in the alveolar socket of tooth no. 46 and splinting tooth no. 38 to teeth nos. 45 and 47; (**O**) autogenous material covered with A-PRF membrane; (**P,Q**) the wounds (places of teeth nos. 38 and 46) were sutured tightly and without tension (sutures 4-0); (**R**) Kinesio tape application.

In anticipation of the surgical procedure, the patient received professional dental prophylaxis, complemented by a regimen of oral rinsing with a 0.1% chlorhexidine solution (Eludril Classic, Pierre Fabre Oral Care, Lavaur, France), twice a day for four days before surgery. This pre-operative hygienic protocol was meticulously followed to significantly diminish the oral microbial population, which is a critical step in preventing infections and promoting optimal surgical outcomes. The patient obtained a complete blood count test before the procedure, which showed no abnormalities. Before surgery, venous blood was collected from the patient (Figure 2A–C; 40 mL of venous blood into four sterile, anticoagulant-free, glass-coated plastic tubes—10 mL each) in a separate office. Blood was taken from the cephalic vein. Then, tubes were immediately centrifuged (All Centrifuge, Scilogex, LLC, Rocky Hill, CT, USA). The time from blood collection to centrifugation cannot exceed 2 min. This is related to the lack of anticoagulant in the blood collection tube and the blood must be centrifuged before it will start coagulating. Centrifugation time was 14 min, at a speed of 1500 rpm. After centrifugation, four fibrin clots were obtained. A-PRF clots were removed from blood tubes and then were dissected by scissors from the red blood cell base at the bottom, 2 mm below the connection between layers. The connection areas were rich in platelets and growth factors [28]. After, A-PRF clots were put in a special PRF box (Quadrostom, Kraków, Poland). One clot was made into an A-PRF membrane, and the rest were mixed with ground tooth no. 46. The A-PRF clots and membranes were rich in growth factors.

The procedure was performed under local anesthesia and given via injection. Mandibular, lingual, and buccal nerves were blocked in the third and fourth quadrants using 4% articaine hydrochloride containing 1:100 000 epinephrine (two ampules per side; Citocartin 100, Molteni Dental s.r.l., Scandicci (Florence), Italy). Then, an envelope incision was made using a no. 15c scalpel blade and mucoperiosteal flaps were raised (Figure 2D–R). Tooth no. 46 was cut, and osteotomy was performed using a round bur (*carbide Lindemann milling cutter HM 408M, Meisinger, Hager & Meisinger GmbH, Neuss, Germany*) mounted on a surgical contra-angle handpiece (WS-92 L, W&H Dentalwerk Bürmoos GmbH, Bürmoos, Austria), at 50,000 rpm with abundant irrigation of 0.9% NaCl. After crown–root separation, the roots were removed atraumatically using ‘luxing periotom‘ (Pol-Intech, Łódź, Poland). Then the tooth was prepared and processed in a Smart Dentin Grinder (Kometa Bio Headquarters, Cresskill, NJ, USA). The enamel, caries, dental filling, and soft tissues were removed from the tooth surface. The tooth was placed in the device, ground, and then the dentin was cleaned. The autogenous dentin graft was mixed with A-PRF clots to achieve a “sticky tooth”.

The triangular incision in the area of tooth no. 38 and retromolar on the left side was made with a no. 15c scalpel blade, and mucoperiosteal flaps were released. Osteotomy was conducted and tooth no. 38 was removed using a minimally invasive approach. In order to not damage the periodontium, the root surface must not be touched while the tooth is being transferred. Tooth no. 38 was transplanted without bone blocking in the place of tooth no. 46. The autogenous material was covered using an A-PRF membrane. Due to the osteotomy, we decided to use the splinting time recommended for jaw fractures, rather than for avulsion. However, after a month, tooth mobility was still increased, so the splint was finally placed on the teeth for 3 months. Stabilization of tooth no. 38 was obtained using a sectional 0.016” stainless steel archwire that was passively bent. The additional wire was first ligated to the brackets on the adjacent teeth and then attached to the transplanted tooth with composite to obtain passiveness. The round stainless steel archwire was chosen because of the large distance between the brackets. 

**Figure 3:**
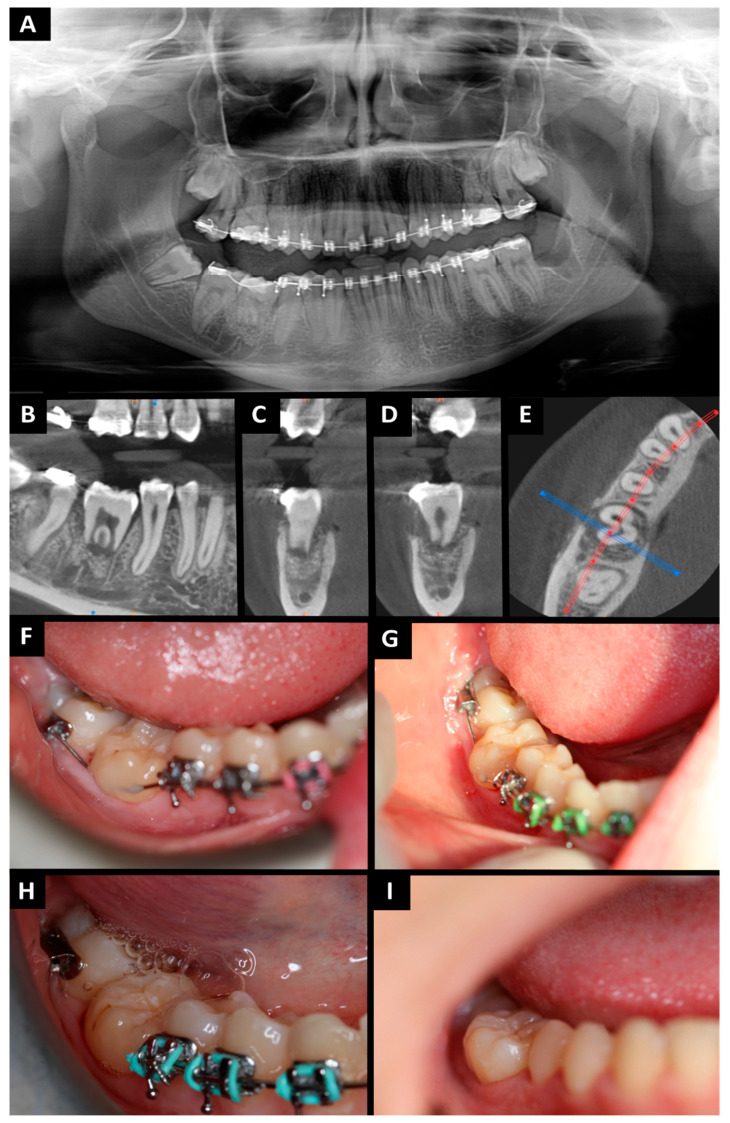
Orthopantomography (**A**) tooth no. 38 in place of tooth no. 46 (immediately after surgery); CBCT after 6 weeks (**B**–**D**): (**B**) pantomography reconstruction—tooth no. 38 in place of tooth no. 46; (**C**) cross-sectional view—mesial root of tooth no. 38; (**D**) cross-sectional view—distal root of tooth no. 38; (**E**) axial view—visible bone healing; (**F**) soft tissue healing after 6 weeks; (**G**) soft tissue healing after 5 months; (**H**) intraoral photography after 6 months; (**I**) intraoral photography after 6 months.

The autotransplanted tooth was excluded from occlusion. The wounds (places of teeth nos. 38 and 46) were sutured tightly and without tension. Absorbed polyglycol sutures 4-0 were used. Hemostasis was achieved. Tooth no. 38 was stitched using metal ligatures. A gauze pad was put on and the patient was instructed to bite on it and hold it for 20 min. To prevent postoperative edema, pain, and trismus, Kinesio tape (KT) was applied [34,35]. A 5 cm wide plaster (cotton, acrylic adhesive, hypoallergenic) was used. KT was divided into four parts, with one common end. The central KT was tightened by 15%. The tape was applied to clean skin and applied from the collarbone (common end) to the zygomatic arch (free ends). A postoperative radiological examination was performed (Figure 3A). 

The patient was prescribed antibiotics (Clindamycin 0.300 g (Dalacin c, Pfizer, Brooklyn, New York, US), every 8 h for 7 days) and analgesics (Nimesulide 0.1 g (Berlin-Chemie, Berlin, Germany), twice daily in case of pain). In the perioperative period, mouthwash with 0.1% chlorhexidine solution (Eludril Classic, Pierre Fabre Oral Care, Lavaur, France) twice a day for 10 days was recommended.

**Figure 4:**
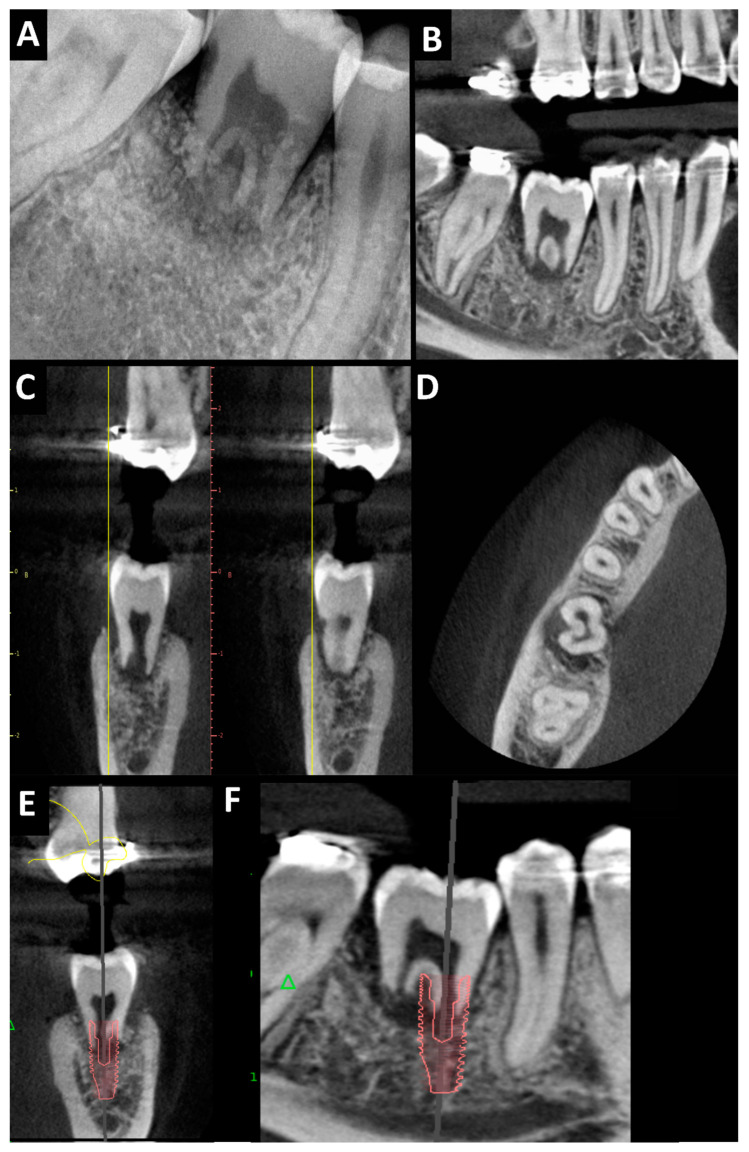
Pulp necrosis and reconstruction of the bone defect—diagnostic imaging one year after the surgical procedure showing cervical inflammatory resorption, unchanged pulp canal dimensions, absent root growth, periapical radiolucency, lack of apical and marginal healing, obtaining interproximal bone with its normal level, and bone presence at the labial and lingual side of the transplanted molar: (**A**) dental X-ray—tooth no. 38 in place of tooth no. 46; CBCT after 1 year—tooth no. 38 in place of tooth no. 46; (**B**–**D**): (**B**) pantomography reconstruction; (**C**) cross-sectional view; (**D**) axial view—visible bone healing; implant planning (**E**,**F**): (**E**) cross-sectional view; (**F**) orthopantomography reconstruction (planed implant: ø 4.2 mm, H 13 mm).

The patient had follow-up visits after 48 h, 7 days, 14 days, 6 weeks, 3 months, 6 months (Figure 3G–I), and 1 year after the procedure (Figure 4). At each visit, the following clinical features were assessed: the sensibility of the pulp on cold stimulus (ethyl chloride) and the depth of gingival pockets. After the surgery, wound healing went well and painlessly. The sutures were removed on day 7. After 14 days and 6 weeks, the condition of the tissues around the transplanted tooth was stable and no inflammation was detected. After three months, there were no signs of inflammation, and the soft tissues were properly healed. The splint was removed. After removing the splint, tooth stability was measured using Periotest (Medizintechnik Gulden e.K., Modautal, Germany; PTV—Periotest value; physiological mobility PTV −08 to +09; I grade of mobility +10 to +19; II grade of mobility +20 to +29; III grade of mobility +30 to +50). The PTV value was +7.1, which corresponds to physiological mobility. Periodontal pocket measurements using the Caroline probe showed the following: mesiobuccal depths of 3 mm, mesiolingual depths of 3 mm, distal–buccal depths of 4 mm, and distal–lingual depths of 4 mm. The pulp sensibility test was not reliable probably due to pulp shock after the procedure. The transplanted tooth was not included in the orthodontic appliance. Six months after the procedure, the patient came for a follow-up visit. The patient did not report any problems in the oral cavity. The intraoral examination showed complete healing of the soft tissue. The pulp sensibility test was inconclusive. The PTV value was +5.8, which is within the limits of physiological mobility. Periodontal pocket measurements showed mesiobuccal depths of 3 mm, mesiolingual depths of 3 mm, distal–buccal depths of 4 mm, and distal–lingual depths of 4 mm. 

After one year, in the clinical examination, there were no signs of inflammation of the soft tissues. The PTV value was +11. Periodontal pocket measurements showed mesiobuccal depths of 3 mm, mesiolingual depths of 3 mm, distal–buccal depths of 4 mm, and distal–lingual depths of 7 mm. A dental X-ray and CBCT were performed (Figure 4A–D) and showed cervical inflammatory resorption, unchanged pulp canal dimensions, absent root growth, periapical radiolucency, and a lack of apical and marginal healing. Due to the pulp necrosis with mobility of the tooth and the presence of resorption, we did not perform root canal treatment of the tooth as an attempt to treat resorption. The tooth was qualified for extraction with immediate implantation. The width and height of the alveolar part of the mandible were correct, and the mesiodistal space between teeth nos. 45 and 47 was preserved, as well as the bone peaks and papillae at teeth nos. 45 and 47 (Figure 4E,F). For this patient, predictable immediate implant placement could have been performed. The patient did not consent to the proposed treatment. Due to the patient’s lack of consent to implant treatment, the transplanted tooth was removed. Healing was good, without complications.

## 3. Discussion

Treating children and adult patients with infraocclusion is difficult. In cases of severe reinclusion of a permanent tooth, it is hard to determine a clear algorithm. Treatment protocols need to be interdisciplinary and patient-centered. An interdisciplinary approach involves specialists from different disciplines of dentistry, such as general dentists, orthodontists, prosthetists, oral surgeons, and periodontists. As a team, they should work collaboratively. In any case of reinclusion, treatment must be individually adapted to the patient. The treatment plan should consider the patient’s age, the degree of immersion, the severity of tooth ankylosis, the positions of the adjacent teeth, profiles, available space, and malocclusion. In the case of partial infraocclusion, the dentist should precisely examine the tooth clinically and radiographically and its tendency to submerge [3]. 

The treatment options are orthodontic extrusion, extraction with space closure, extraction with tooth replacement (implant or bridge), decoronation with delayed implantation, and extraction with autotransplantation of the immature third molar. Positive results may be obtained by extrusion using orthodontic high forces [34,35,36,37]. To make orthodontic extrusion easier, an additional surgical luxation of the permanent tooth may be performed before the orthodontic traction [3,36,37,38,39]. 

In our case, orthodontic extrusion was ineffective. It could be due to the collision of cortical bone and enamel covering the crown resulting from the inability to remove enough bone during the surgery to create a clear path for extrusion. Moreover, the tooth experienced dilaceration of the mesial root, which was an additional limitation for orthodontic tooth movement [40].

In the presence of caries, inflammation, abscesses, significant inclination of adjacent teeth, and severe submerging of a tooth, extraction should be performed. The right moment for the removal of the tooth has not been specified and requires further research. According to studies, the extractions of maxillary first permanent molars performed between the ages of 8 and 10.5, and the removal of mandibular ones performed in patients aged 8 to 11.5 years, provide good clinical results for spontaneous space closure. The mesialization of second permanent molars allows for improved bone conditions of the alveolar bone (in line with their eruption). Further movement could be performed with orthodontic forces following the eruption [41].

Removing a tooth with infraocclusion is a difficult procedure due to the inclination of the adjacent teeth, dilaceration of roots, and immersion of the crown. It is necessary to consider the presence of adjacent structures—the maxillary sinus and inferior alveolar nerve canal (IANC). During the procedure, the maxillary sinus may be opened or the neurovascular bundle in the IANC canal may be damaged. The first complication may contribute to the formation of an oro-antral communication or fistula, inflammation, and empyema of the maxillary sinus. Damage to IANC may lead to temporary or permanent sensory disturbances in the lower lip (Vincent’s sign) and bleeding. Surgical extraction of a reincluded tooth requires crown–root separation of the tooth and, at times, osteotomy. This is associated with large bone loss in the vertical and horizontal dimensions of the mandibular body. This may even result in a pathological fracture of the mandible [1,4,42,43]. Space closure may be impossible due to expected bone defects after extraction [44]. Extraction and implantation would require extensive and unpredictable bone regeneration. Concomitantly, making a bridge would involve healthy tooth preparation.

Alternative treatments for such patients can be decoronation or autotransplantation. Decoronation involves cutting off the embedded tooth 1.5–2.0 mm below the edge of the alveolar ridge and then suturing the wound with a mucoperiosteal flap, without contact with the oral cavity. Only teeth without pulp inflammation or periapical lesions can be subjected to this treatment. Decoronation affects further growth of the alveolar bone. This is related to the formation of new periodontal fibers over the decorated tooth and pulling it through the adjacent teeth [45,46,47]. In this case, decoronation would not be a good solution because of the large bone defect. 

Autotransplantation is a dental procedure that involves transplanting a tooth from one position to another within the same patient. This technique is used to replace missing or damaged teeth with the patient’s own healthy teeth, ensuring compatibility and reducing the risk of rejection. It can be performed simultaneously with the removal of the causative tooth, or in two stages (first stage—removal of the reincluded tooth, second stage—autotransplantation procedure). A donor site must always be prepared. This can be conducted using a replica of a donor tooth or a free hand. The tooth replica is made via 3D printing or milling. The future socket must be well adapted to the donor. Then the transplanted tooth should be fixed. Sutures or semi-flexible splints can be used. The stage of root development influences the success rate of autotransplantation. Teeth with root lengths of one-half or three-quarters were found to be the most successful. According to the classification by Moorrees, Fanning, and Hunt, these are stages R1/2 and R3/4. Moreover, teeth with completed root development require root canal treatment after the surgery due to doubtful revascularization. The transplant ensures bone preservation, and in cases of early surgery, with its coverage of soft tissue and subsequent spontaneous eruption, it allows for an increase in the amount of bone [48,49,50,51,52]. Autotransplantation is a proven treatment method, and premolars are the preferred donor teeth in the anterior region [53]. A very recent study evaluating 910 transplanted premolars showed that the overall survival and success of autotransplanted immature premolars after an observation period of 10 years was 99.8%. The 10-year survival and success rates when premolars with fully developed roots were transplanted in the anterior region in adolescents were 100% and 96.3%, respectively. In adults, the 10-year survival/success rate was 87.5% [54]. Molar transplants have a higher failure rate, increased risk of ankylosis, abnormal mobility, unacceptable pocket depth, and root resorption compared with premolars. The considered explanation is that molars are more difficult to remove atraumatically even when taking care of every aspect [55,56]. Due to the size of the space, incomplete root development of the third molars, and the posterior crowding around the third molars, the opposite third molar was selected as the donor.

Platelet-rich fibrin (PRF) is an autogenous matrix obtained from the patient’s platelet concentrate. It works by accelerating the healing and improving its quality. PRF demonstrates an absence of adverse antigenic reactions following graft implantation and throughout the integration process. This emphasizes PRF’s biocompatibility and its role in promoting a favorable environment for tissue regeneration without eliciting immune-mediated responses. The autologous biomaterial has a tetramolecular structure and is composed of a fibrin matrix that traps platelets, leukocytes, cytokines, and circulating stem cells. Growth factors, including platelet-derived growth factor (PDGF-β), fibroblast growth factor (FGF), transforming growth factors (TGF-⍺ and β1), vascular endothelial growth factor (VEGF), insulin-like growth factor, leukocytic cells and their cytokines (interleukin 1β, IL-6, IL-4), tumor necrosis factor α (TNF-α), bone morphogenetic proteins (BMPs such as BMP-2), and matrix metalloproteinases (MMPs such as MMP-9) are enmeshed within the fibrin matrix. It is proven that there are platelets present in the peripherals of the clot in A-PRF. The difference in the processing can be responsible for more optimized, longer-lasting, and more even distribution and release of growth factors from A-PRF to the surrounding tissues, affecting tissue regeneration and maturation. The distribution of lymphocytes, macrophages, and stem cells is greater in the proximal part of the clot, whereas neutrophils are located mainly in the distal part [11,12,13,14,15,16]. 

A-PRF is utilized in many fields of dentistry such as oral surgery, implantology, periodontology, and endodontic wound healing treatment. PRF can be administered to preserve a socket after tooth extraction, promote healing of infrabony defects, support bone regeneration during implant placement, and ensure adequate osseointegration and soft tissue healing. It can be used alone or along with a bone graft [15,17,18,19,20,21,22,23,24]. The compressed A-PRF membrane acts as a barrier membrane. Mixing injectable platelet-rich fibrin (I-PRF) with the bone graft granules results in the formation of a sticky bone [17,23,25]. Mixing I-PRF with dentine graft granules results in the formation of a “sticky tooth” [23,26].

Autogenous tooth bone graft material has a similar structure to bone. Dentin, which constitutes the main mass of the tooth, is an acellular, collagen-rich matrix without blood vessels. Bone, on the other hand, is a cellular, vascularized tissue. However, the chemical composition of dentin closely resembles human bone tissue. It consists of 20% of the organic part (the main ingredient of which is type I collagen, 70% of the inorganic supporting part (mainly hydroxyapatite), and 10% water. In bones, the same ingredients are distributed as follows: 25%, 65%, and 10% [27]. Dentin comprises type I collagen (90%), biopolymers, citrate, lipids, and non-collagenous proteins. Moreover, dentin and cementum contain several growth factors that induce bone formation by stimulating osteogenic cell activity and osteoconductive properties. The bone morphogenetic proteins (BMPs) contained in the dentin are responsible for its osteoinductive capacity. The presence of other growth factors, such as TGF-beta, FGF, PDGF, and EGF, has also been reported. Osteoprogenitor and osteoconductive cells, growth factors, and mechanical stabilization are necessary to achieve bone formation [28,29,30,31,32,33].

Numerous studies have documented the efficacy of platelet-rich fibrin in promoting bone regeneration in graft areas. When platelet products are added to different kinds of graft materials, a more predictable outcome is derived after bone augmentation [13,15,16,17,18,19,20,21,22,23,38]. The literature includes a few studies using only PRF or graft materials with different characteristics combined with PRF. PRF in addition to the particulate autogenous bone graft may favor the formation of new bone and PRF keeps the graft particles together. Based on our results, applying PRF with dentine matrix to the bone defects may accelerate graft healing and shorten the rehabilitation time. The hemostatic effect of the PRF (stopping bleeding in a short time) is important for keeping graft particles together in the bone defects [57,58].

The use of the autogenous dentin graft and platelet-rich fibrin is not a standard procedure in bone regeneration. Both materials have already been tested in an animal model and in a few patients. The studies have shown good new bone formation around implants or in post-extraction sockets. Therefore, it is an encouraging method to use one’s own tissues, which are non-immunogenic and non-allergenic, for this purpose [59,60].

In our study, we observed exceptionally favorable bone conditions post-procedure, successfully preserving both the height and width of the alveolar ridge in the mandibular region. This outcome was achieved through a novel approach diverging from conventional tooth extraction methodologies, which typically do not incorporate additional procedural interventions. Such traditional methods often result in significant vertical and horizontal bone loss, leading to the diminution of bone peaks, and complicating, if not rendering impossible, subsequent bone regeneration efforts. Moreover, conventional techniques fail to maintain the ideal positioning of gingival papillae, adversely affecting the aesthetic harmony between gingival tissues and teeth—the so-called red and white aesthetics. Our research emphasizes the efficacy of employing autogenous agents in dental surgery, offering a minimally invasive alternative that effectively mitigates the risk of immunological or allergic reactions, given the absence of xenogeneic or allogeneic materials. This approach not only facilitates the preservation of alveolar bone integrity but also opens the door to the potential use of autotransplantation as a viable alternative to traditional implant treatments. In instances of treatment failure, ADG emerges as a commendable augmentation material. This technique proves particularly beneficial in socket preservation when all alveolar walls are intact. Conversely, in scenarios where alveolar wall defects are present, ADG, supplemented with a membrane, serves as an effective strategy for alveolar ridge preservation. Notably, an autogenous PRF membrane, prepared through a specific pressing technique, can be utilized to enhance this process. Our findings advocate for a paradigm shift toward minimally invasive, autogenous approaches in dental surgery, highlighting their potential to significantly improve patient outcomes while minimizing risks associated with traditional bone grafting and implantation procedures.

One of the primary challenges encountered in our study stemmed from pre-existing bone defects due to tooth reinclusion and abnormal growth of the alveolar part of the mandible around this tooth. Such conditions inherently complicate the procedural outcomes and may limit the applicability of our findings to cases with similar preoperative conditions. The extraction of the tooth was notably difficult, necessitating crown–root separation followed by osteotomy, which inadvertently introduced a small secondary bone defect. This complication added a layer of complexity to the procedure, potentially affecting the healing process and the overall success of the autotransplantation. The roots of teeth 38 and 48 were at similar stages of development—stage F, according to the Demirjian method for dental age estimation, crown shape, and size [61]. Concomitantly, the right mandibular third molar was more covered with bone, which made its extraction seem to be more traumatic. Additionally, we decided to leave this tooth in case the autotransplantation failed and to attempt orthodontic mesialization of teeth nos. 47 and 48. Although the donor’s qualification was deemed appropriate based on the Moorrees, Fanning, and Hunt classification (R1/2 stage of root development), the significant size disparity between the donor tooth (38) and the recipient site posed a considerable challenge. The recipient site was not prepared as it was larger than the donor tooth. The resulting gap necessitated the use of the autogenous dentin graft, yet the extensive space raised concerns regarding the adequacy of time for the transformation of dentin into bone. 

External inflammatory root resorption is one of the complications after autotransplantation. It is associated with massive damage to the cementum and concomitant infection of the canal system. It may occur anywhere along the length of the canal and in the periapical area [62]. The inflammatory resorption that occurred in our case was present in the cervical area of the donor tooth. It was most probably because of necrosis and long-standing infection of the pulp. As the International Association of Dental Traumatology (IADT) guidelines for immature avulsed teeth do not recommend routine endodontic treatment after replantation, especially if it occurs immediately after tooth avulsion, we also did not undertake such treatment, hoping for revascularization and further root development. The relationship between root development and pulp necrosis is very strong [63]. One of the reasons for the failure of our treatment could be an inappropriate bed for the donor and the lack of preparation. The successful healing of the autotransplanted tooth depends, among other things, on the shape and the site of the recipient socket, as well as on the vascularity of the recipient bed. Transplantation of a tooth into a socket with regenerative tissues could potentially favor healing and reduce the risk of inflammatory root resorption [64]. Surgical preparation of the recipient bed and leaving it for a few days to start the regeneration of tissues prior to transplantation seems to be worth considering as one of the methods to prevent resorption. Moreover, the entry of saliva-borne bacteria into the tooth socket and thermal trauma, despite abundant cooling, could have affected the healing process. 

In the presented case report, the recipient site was not previously prepared, because it was larger than the donor’s tooth and the transplantation was made immediately after the extraction. Moreover, since tooth no. 46 was infraoccluded, its removal was a complex procedure, leaving much larger bone defects compared to the extraction of a tooth properly positioned in the dental arch.

Another issue worth considering in the context of tissue healing is the splinting of teeth after autotransplantation. A step-by-step guideline for autotransplantation indicates that seven days of suture fixation of a donor tooth should be sufficient to achieve stability. However, regarding the high mobility of the transplant and large defects of the bone in the recipient site, like in our case, the stabilization should be extended with a wire-composite splint for several months [65]. Studies reported that prolonged, rigid fixation may cause disturbances in pulp revascularization, resulting in tooth necrosis and inflammatory root resorption. That is why some authors suggest that in cases of both large bone defects and a lack of stability of the transplanted tooth, a two-phase surgical procedure after regeneration of the alveolar socket should preferably be considered for long-term splinting (4 weeks and more) [66]. 

Managing the external inflammatory resorption involves root canal treatment carried out in a way that encourages the healing and repair of hard tissue and PDL. The treatment protocol includes the use of intracanal dressings with corticosteroid/antibiotic (CS-AB) paste alone and then the mixtures of CS-AB with calcium hydroxide. Once tissue healing occurs, the root canal filling can be conducted [62]. However, if the tooth structure is insufficient and the long-term prognosis is poor, then the tooth should be extracted, as in the presented case report.

The systematic use of antibiotics after surgical tooth extraction—as a way of preventing infection and alveolar osteitis—is a concept that is still being studied and discussed in the literature. It is believed that the third molar surgery should be associated with the administration of antibiotics [7]. However, considering increasing antimicrobial resistance, the prophylactic use of systemic antibiotics remains controversial. Therefore, other forms of antibiotic administration and preventive measures are being sought that will be effective in reducing the risk of inflammatory postoperative complications. Various preventive procedures have been proposed in the literature. One of them is preoperative and postoperative mouth rinsing with 0.12% chlorhexidine, which reduces the occurrence of alveolar osteitis. Other agents used to minimize the risk of postoperative infection are antibiotics placed into the socket after tooth extraction (local drug delivery). They can be in the form of tetracycline or clindamycin-impregnated Gelfoam [8]. Oxytetracycline-impregnated intra-socket gauze drain was also investigated as an alternative to systemic antibiotics; however, this management strategy needs more and better-designed studies [9] In the presented case, the patient was given systemic antibiotics and advised to rinse his mouth with a 0.1% chlorhexidine solution before and after the surgical procedure. The study also explores the feasibility of immediate implant placement, which was deemed predictable given the suitable width and height of the alveolar mandible and the preservation of mesiodistal space and anatomical landmarks. However, the patient’s decision against opting for implant–prosthetic treatment limits the direct comparison of outcomes between the autotransplantation method and alternative implant strategies. A noteworthy limitation of our study is the patient’s reluctance to pursue the proposed implant–prosthetic treatment. This decision restricts our ability to assess the long-term efficacy and patient satisfaction with the autotransplantation approach compared to other available treatment modalities.

Early and accurate diagnoses are very important in the effective treatment of infraocclusion. However, this article broadens the horizon to include the latest advances in diagnosis and treatment, offering new insights into potential treatments. The described clinical case shows the role of growth factors and the processed tooth as supporting tissues during autotransplantation, maintaining the continuity of the dental arch, easier prosthetic reconstruction, and not disturbing the occlusal plane. Future research should focus on large cohort studies using autologous tissues as the gold standard for soft tissue and bone regeneration. Such tissues are identical to the human body and do not cause allergies or biological reactions. Currently, there is an attempt to imitate nature and biomimetic treatment. This is a new direction of research that requires further development. More patients are interested in such solutions due to their lives and religious principles.

Like any treatment technique, the presented method has some limitations. It should not be used in immunocompetent patients, with uncontrolled systemic diseases, and in oncological patients. This is important because in such situations, healing in patients is unpredictable, and such treatment may end in failure. The limitation of this article is that the treatment was only performed on one patient. Future research directions should focus on large cohort studies and the use of autotransplantation of developing teeth, ADG, and A-PRF for bone regeneration in tooth eruption disorders and tooth replacement in severe molar incisor hypomineralization, as well as in more frequent clinical situations, like complications of severe caries. The case report described in this article shows new directions for research and development in dentistry. 

## 4. Conclusions

The presented case of autotransplantation of a mandibular left third molar to replace the mandibular right first molar with a severe infraocclusion highlights the difficulties and complications associated with this procedure. The status of the alveolar bone at the recipient site is among the factors that are associated with the success of tooth autotransplantation. Both the buccopalatal and vertical height of the bone, as well as the good quality of the bone, promote the healing of the donor’s tissues. Severe tooth infraocclusion itself leads to significant bone defects, which may be additionally enlarged during the complicated extraction of the reincluded tooth. Great caution should be advised when planning autotransplantation procedures at the site after extraction of the severely submerged tooth. In such cases, bone grafting procedures and bone regeneration techniques appear to be necessary prior to tooth transplantation. This is the first article discussing the usage of autogenous dentine grafts and advanced platelet-rich fibrin in bone regeneration during autotransplantation. However, there are some limitations to our paper. The follow-up was one year, and the procedure ultimately failed. Therefore, we see a great need for clear transplantation protocols for teeth in sites with inadequate alveolar bone status. 

## Data Availability

The data presented in this study are available on request from the corresponding author.

## Abbreviations

A-PRF	advanced platelet-rich fibrin
ADG	autogenous dentine graft
ATBG	autogenous tooth bone graft
BMP-2	bone morphogenic factor 2
BMP-7	bone morphogenic factor 7
CBCT	cone-beam computed tomography
FGF	fibroblast growth factor
I-PRF	injective platelet-rich fibrin
IANC	inferior alveolar nerve canal
IOTN	Index of Orthodontic Treatment Need
IL-1β	interleukin 1β
IL-4	interleukin IL-4
IL-6	interleukin IL-6
KT	Kinesio tape
L-PRF	leukocyte-platelet-rich fibrin
PD-EGF	platelet-derived epidermal growth factor
PDGF	platelet-derived growth factor
PRF	Platelet-rich fibrin
PRP	Platelet-rich plasma
TGF	transforming growth factor
TNFα	tumor necrosis factor α
TGFβ-1	tumor necrosis factor β-1
TSP-1	thrombospondin-1
VEGF	vascular endothelial growth factor
